# Sperm Injection at the Para‐Polar Body Site in Piezo‐Intracytoplasmic Sperm Injection Improves Subsequent Early Development of Bovine Embryos

**DOI:** 10.1002/rmb2.12660

**Published:** 2025-09-01

**Authors:** Shiori Ashibe, Yoku Kato, Sarentonglaga Borjigin, Yoshikazu Nagao

**Affiliations:** ^1^ University Farm Faculty of Agriculture, Utsunomiya University Tochigi Japan

**Keywords:** bovine, injection site, ooplasmic cell membrane, piezo pulses, piezo‐ICSI

## Abstract

**Purpose:**

In piezo‐ICSI, the first polar body (PB) of a metaphase II (MII) oocyte is generally oriented in the 6 or 12 o'clock position relative to sperm injection at 3 o'clock. However, the ooplasmic cell membrane may be damaged during drilling of the zona pellucida by piezo pulses. Here, we tested a new piezo‐ICSI method in which the PB is set at the 2 or 4 o'clock position, so that zona drilling is performed through the widest position in the perivitelline space (para‐PB piezo).

**Methods:**

The effect of piezo pulse position was evaluated by propidium iodide staining and cytoplasmic Ca^2+^ levels. The effect of injection site on integrity and movement of the meiotic spindle was evaluated by microscopy and an Oosight imaging system. Bovine oocyte survival rate, developmental competence, and chromosomal integrity at the blastocyst stage were compared between para‐PB piezo and conventional piezo groups.

**Results:**

Piezo pulses may induce slight changes in the ooplasmic cell; our piezo‐ICSI method can improve oocyte survival by minimizing damage to the ooplasmic cell membrane during zona drilling and consequently increasing the number of embryos available for transfer.

**Conclusions:**

Para‐PB site piezo‐ICSI is an improvement on current ICSI technology for animal and human reproduction.

## Graphical abstract

In piezo‐ICSI, the first polar body (PB) of a metaphase II (MII) oocyte is generally oriented in the 6 or 12 o'clock position relative to sperm injection at 3 o'clock. Here, we tested a new piezo‐ICSI method in which the PB is set at the 2 or 4 o'clock position, so that zona drilling is performed through the widest position in the perivitelline space (para‐PB piezo). Piezo pulses may induce slight changes in the cell membrane; our piezo‐ICSI method can improve oocyte survival by minimizing damage to the cell membrane during zona drilling and consequently increasing the number of embryos available for transfer.

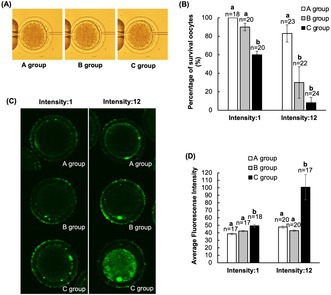

## 1 Introduction

Intracytoplasmic sperm injection (ICSI) has been successfully used to produce live offspring in many species, including livestock and humans [1]. There are two main methods of ICSI: conventional ICSI (c‐ICSI) and piezo‐assisted ICSI (piezo‐ICSI). In c‐ICSI, a micropipette with a spike is used to penetrate the ooplasmic cell membrane, aspirate cytoplasm into the micropipette, and then inject the sperm into the recipient oocyte. In piezo‐ICSI, a micropipette with a flat tip is used to penetrate the ooplasmic cell membrane via a piezo pulse and to inject the sperm into the oocyte [2]. Compared with c‐ICSI, piezo‐ICSI significantly increases the rate of fertilization of surviving oocytes after sperm injection in humans [3, 4] and cow [5]; thus, piezo‐ICSI is considered less invasive to the oocyte. Although piezo‐ICSI has been used in clinical assisted reproductive technology, there is limited information on this method. Hiraoka et al. [6] reported that reducing the thickness of the micropipette wall causes less damage to the oocyte and improves survival and fertilization rates in piezo‐ICSI. To our knowledge, however, no studies have explored the optimal site of sperm injection in piezo‐ICSI.

In general, in both c‐ICSI and piezo‐ICSI, the first polar body (PB) of a metaphase II (MII) oocyte is oriented in either the 6 or 12 o'clock position relative to injection of the sperm at the 3 o'clock position. This is based on the assumption that the meiotic spindle is located near the PB and that sperm injection at 3 o'clock will avoid damage to this structure [7]. In piezo‐ICSI, however, it is possible that the ooplasmic cell membrane might be damaged during the drilling of the zona pellucida with piezo pulses, resulting in a lower rate of successful development to the blastocyst stage. Certainly, Rienzi et al. reported that, in c‐ICSI, aspiration of cytoplasm into a micropipette can disrupt the ooplasmic cell membrane, which may damage the meiotic spindle in the process [8]. Using a spindle visualization system, Uchiyama et al. [9] further observed that spindle damage during c‐ICSI leads to multicentric pronucleus formation. On the other hand, the risk of spindle damage in piezo‐ICSI may be low because the ooplasmic cell membrane is broken by piezo pulses.

The objective of this study was to determine the effectiveness of a new piezo‐ICSI method in which the PB is set at the 2 or 4 o'clock position. We tested our method in bovine oocytes as a model for human oocytes [10]. We performed zona pellucida drilling and sperm injection through the para‐PB site, which is the widest position in the perivitelline space, and examined how piezo pulses during zona drilling affected the ooplasmic cell membrane, the meiotic spindle, and subsequent embryonic development.

## 2 Materials and Methods

### 2.1 Ovary Collection and Preservation

Bovine ovaries were obtained from a slaughterhouse, maintained at 15°C–20°C, and transported to the laboratory in saline solution supplemented with 0.1% antibiotics and antimycotics (AB; Invitrogen, Carlsbad, CA, USA). The transport time from the slaughterhouse to the laboratory was approximately 1 h.

### 2.2 Oocyte Collection and In Vitro Maturation

Oocyte collection and in vitro maturation were carried out as previously described [11]. In short, cumulus–oocyte complexes were aspirated from follicles of 2–6 mm in diameter using a 20‐gauge needle attached to a 5‐mL syringe. Only good‐quality oocytes surrounded by three or more layers of compact cumulus cells and with an evenly granulated cytoplasm were used for experiments. They were washed and cultured in 50‐μL drops of modified TCM‐199 covered with mineral oil (M8410, Sigma–Aldrich) in a culture dish (Falcon351007, Becton, Dickinson and Company, Franklin Lakes, NJ, USA) for 22–25 h at 39°C under 5% CO_2_ and humidity. Modified TCM199 comprised HEPES‐buffered medium 199 (No. 12340, Invitrogen) supplemented with 0.1% (w/v) polyvinyl alcohol (PVA; P8136, Sigma‐Aldrich), 0.5 mM sodium pyruvate (Nacalai Tesque, Tokyo, Japan), 1% AB, 0.02 AU/mL of FSH (Antrin, Kyoritsu Seiyaku, Tokyo, Japan) and 1 μg/mL of estradiol‐17 β (E2758, Sigma‐Aldrich).

### 2.3 Intracytoplasmic Sperm Injection, Artificial Activation, and Embryo Culture

Intracytoplasmic sperm injection was performed as described previously [12, 13]. In brief, frozen–thawed semen was washed with and then incubated in Brackett and Oliphant medium (BO) [14] supplemented with 0.05% (w/v) polyvinyl alcohol (PVA; P8136, Sigma–Aldrich) and 1% AB (BO‐PVA) for 0.5–3 h at 39°C under 5% CO_2_ with humidity. After maturation of cumulus–oocyte complexes, cumulus cells were thoroughly dispersed with 0.1% hyaluronidase (Sigma Chemical Co.) and removed by vortexing for 7 min. Denuded oocytes with a visible first PB were selected and cultured in modified TCM199 for approximately 1 h at 39°C under 5% CO_2_ and 5% O_2_ with humidity.

Spermatozoa were immobilized by applying several piezo pulses (Cell Tram Vario, PiezoXpert, Eppendorf, Germany) and then singly injected into each oocyte with a minimal amount of medium. Sham injections were performed in a similar manner. The oocytes (ICSI and Sham injection) were cultured in modified synthetic oviduct fluid supplemented with 0.05% (w/v) PVA and 1% AB (SOF‐PVA) with BSA (SOF‐BSA) [11] for 3 h, and the zygote survival rate was evaluated. The oocytes were then treated with 7% ethanol in TCM‐199 containing 1 mg/mL of polyvinylpyrrolidone (P0930, Sigma Chemical Co.) for 3 min at 39°C under 5% CO_2_ for oocyte activation [12]. Seven days after sperm injection, cleavage (≥ 2‐cell) and development to the blastocyst stage were examined under a stereomicroscope (×60 magnification).

### 2.4 Experimental Design

We conducted three experiments to optimize the injection site in the piezo‐ICSI method. In Experiment 1, we examined the effect of piezo pulse position on the subsequent development of the zygote. We performed the drilling of the zona pellucida with a long (Group A) or short (Group B) distance between the cell membrane and the edge of the micropipette, or with the edge of the micropipette touching the cell membrane (Group C). We then applied piezo pulses for 0.5 s with intensity 1 or 12. In Experiment 2, we examined the effect of the injection site on the integrity of the meiotic spindle in MII oocytes. We performed ICSI with the first PB of an MII oocyte in either the 6 or 12 o'clock position (Conventional piezo group), or 2 or 4 o'clock position (Para‐PB piezo group). As a control, we performed Sham injection in a Conventional piezo group and a Para‐PB piezo group. Movement of the oocyte meiotic spindle during injection was monitored by an Oosight imaging system (Hamilton Thorne, USA). In Experiment 3, we evaluated the effect of the injection site on the survival rate of the oocyte and on developmental competence and chromosomal integrity at the blastocyst stage.

### 2.5 Evaluation of Ooplasmic Cell Membrane Damage

At 24 h after drilling of the zona pellucida, oocytes were cultured for 5 min in SOF‐PVA with 100 μL/mL of propidium iodide (PI; LIVE/DEAD Sperm Viability Kit, Molecular Probes Inc., USA). They were then washed in 1 × Dulbecco's Phosphate Buffered Saline (142200‐075, Gibco, Grand Island; NY, USA) supplemented with 1% (w/v) PVA. Stained oocytes were mounted on a glass slide with 50% (v/v) glycerol and examined under a fluorescence microscope (OLYMPUS, Tokyo). Oocytes with staining of both the first PBs and nuclei were judged to have died owing to cell membrane damage.

Calcium levels in the oocytes were measured by Fluo‐4 AM (F311, Dojindo Laboratories, Japan) as follows. Immediately after drilling of the zona pellucida, oocytes were cultured in SOF‐PVA with 5 μM Fluo‐4 AM for 1 h. They were then placed on a glass dish with SOF‐PVA and examined under a fluorescence microscope (OLYMPUS; excitation, 494 nm; emission, 516 nm). Images were analyzed using FL10‐ASW3.1 software (OLYMPUS); the intensity of fluorescence in each oocyte was used as an indicator of Ca^2+^ level.

### 2.6 Evaluation of Meiotic Spindle Damage

At 18 h after sham injection, oocytes were fixed on a slide with 3:1 acetic acid: ethanol and stained with 1% orcein. Oocytes with scattered spindles were evaluated as abnormal. To assess the movement of the meiotic spindle during sperm injection, oocytes were analyzed during sham injection using an Oosight imaging system (Hamilton Thorne, USA).

### 2.7 Evaluation of Chromosomal Integrity at the Blastocyst Stage

Chromosomes were counted as previously described [15]. In brief, blastocysts were washed and placed in 0.4 mL of 1% sodium citrate solution for 15 min and fixed by adding 0.03 mL of fixative (1:1 acetic acid:ethanol). Blastocysts were placed onto a glass slide, covered with a small drop of acetic acid to separate each blastomere, and then immediately re‐fixed with several drops of fixative. After drying completely, chromosomes in the blastocysts were stained with 2% Giemsa solution for 15 min, counted, and classified as normal (60, 2*n*) or abnormal (30, *n*; or 90, 3*n*).

### 2.8 Data Analysis

Values are reported as mean ± standard deviation (SD) or standard error of the mean. Differences in survival rate determined by PI and in Ca^2+^ levels were analyzed by analysis of variance (ANOVA) with Fisher's PLSD test. Differences in chromosomal integrity, spindle integrity, survival rate, cleavage rates, and blastocyst rates were analyzed by *χ* ^2^ tests. In all tests, values were considered to be significantly different when *p* < 0.05.

## 3 Results

### 3.1 Effect of Piezo Pulse on the Ooplasmic Cell Membrane and Subsequent Development

**FIGURE 1:**
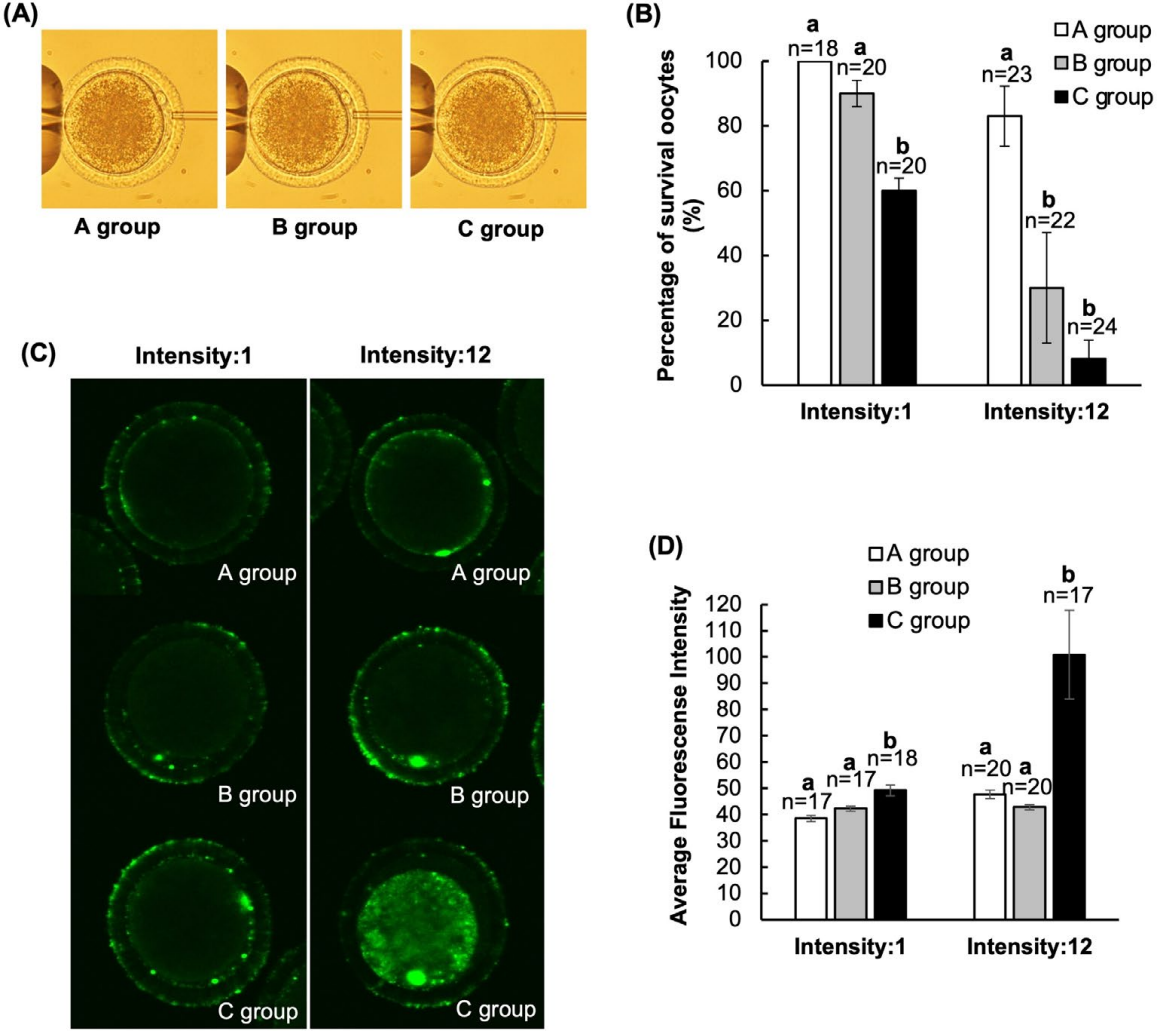
Effect of piezo pulse position on the ooplasmic cell membrane and subsequent zygote development. (A) Distance between the cell membrane and micropipette edge during piezo pulsing. Group A, long distance; B, short distance; C, touching. (B) Percentage of survival rate of the oocytes after micropipette injection. *n*, number of oocytes examined; different letters indicate a significant difference between groups (*p* < 0.05). Data are the mean of four experiments; error bars represent the standard error. (C) Fluo‐4 AM staining of oocytes. (D) Fluo‐4 AM fluorescence as an indicator of intracellular Ca^2+^ levels in oocytes. *n*, number of oocytes examined; different letters indicate a significant difference between groups (*p* < 0.05). Data are the mean of three experiments; error bars represent the standard error.

First, we examined the effect of the position of the micropipette during the application of the piezo pulse on the ooplasmic cell membrane and subsequent development (Figure 1). The survival rate of the oocytes decreased as the distance between the micropipette edge and the ooplasmic cell membrane became smaller. (Intensity 1: Group A, 100%; Group B, 90.0%; Group C, 60.0%. Intensity 12: Group A, 82.6%; Group B, 27.3%; Group C, 8.3%).

In addition, the cytoplasmic levels of Ca^2+^ in the oocytes, as indicated by Fluo‐4 AM fluorescence, increased as the distance between the micropipette edge and ooplasmic cell membrane decreased (Intensity 1: Group A, 38.5 ± 1.2 au; Group B, 42.3 ± 1.1 au; Group C, 49.2 ± 2.0 au. Intensity 12: Group A, 47.7 ± 1.6 au; Group B, 42.8 ± 1.0 au; Group C, 100.9 ± 16.9 au), suggesting increased Ca^2+^ influx and reduced cell membrane integrity.

### 3.2 Effect of Injection Site on Integrity and Movement of the Meiotic Spindle

**FIGURE 2:**
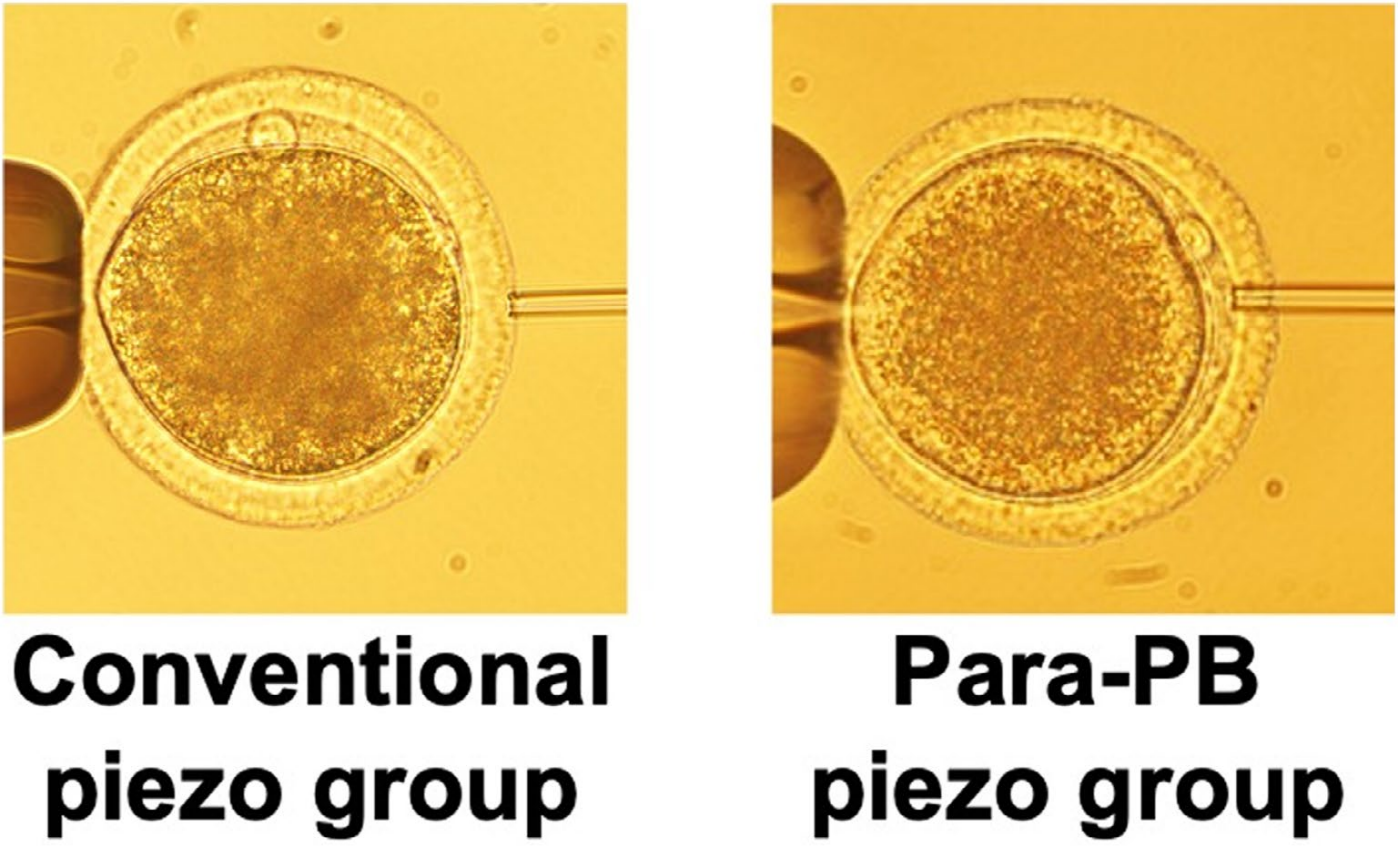
Position of the first polar body (PB) of a metaphase II (MII) oocyte in sperm injection. Conventional piezo group: 6 or 12 o'clock position, corresponding to the narrowest position in the perivitelline space. Para‐PB piezo group: 2 or 4 o'clock position, corresponding to the widest position in the perivitelline space.

**FIGURE 3:**
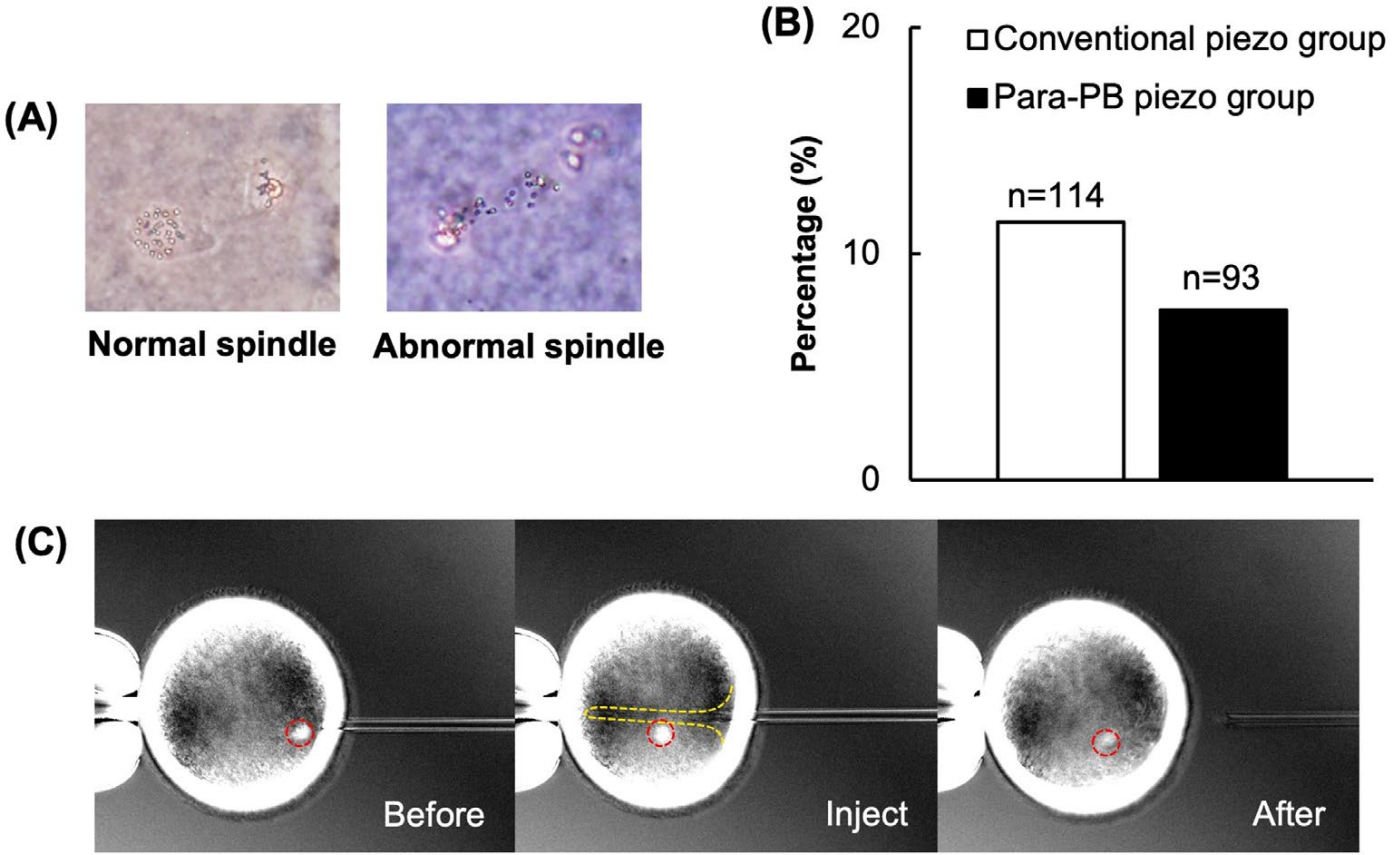
Effect of injection site on the meiotic spindle in MII oocytes. (A) Images of normal and abnormal meiotic spindles after sham injection. (B) Percentage of abnormal spindles in oocytes after conventional piezo‐ICSI and para‐PB piezo‐ICSI. *n*, number of oocytes examined. Data are the mean of four experiments. (C) Movement of the meiotic spindle during micropipette injection. Red dashed line shows meiotic spindle; yellow dashed line shows ooplasmic cell membrane. The images show that the meiotic spindle may escape the micropipette tip if it is pushed away by the ooplasmic cell membrane during micropipette injection.

Next, we examined the effect of the injection site on the meiotic spindle in MII oocytes. We compared the effects of sperm injection relative to the first PB in the 2 or 4 o'clock position (Para‐PB piezo group) with those of conventional ICSI with the first PB in either the 6 or 12 o'clock position (Conventional piezo group) (Figure 2). There were no significant differences in the rate of meiotic spindle abnormalities between the para‐PB piezo group (11.4%) and the conventional piezo group (7.5%) (Figure 3A,B). Observation of the meiotic spindle during sham injection by the Oosight imaging system showed that during micropipette injection, the meiotic spindle was pushed aside by the ooplasmic cell membrane (Figure 3C).

### 3.3 Effect of Injection Site on Developmental Competence at the Blastocyst Stage

Lastly, we examined the effect of injection site on the survival rate of the oocyte, developmental competence, and chromosomal integrity at the blastocyst stage (Table 1). The survival rate was significantly higher in the para‐PB piezo group (90.1%) than in the conventional piezo group (75.0%, *p* < 0.05). However, there were no significant differences in blastocyst rate or chromosomal integrity between the para‐PB piezo group and the conventional piezo group (24.0% vs. 20.7% and 75.0% vs. 72.7%, respectively).

**TABLE 1:** Effect of injection site on survival rate, blastocyst rate, and chromosomal integrity of embryos.

Injection site	No. survival rates (%)	No. cleaved embryos (%)	No. blastocyst (%)	Chromosomal integrity (%)
Conventional piezo group	69/92 (75.0) a	51/92 (55.4)	19/92 (20.7)	8/11 (72.7)
Para‐PB piezo group	109/121 (90.1) b	75/121 (62.0)	29/121 (24.0)	9/12 (75.0)

*Note:* Different letters indicate a significant difference between groups (*p* < 0.05). Data are the mean of five experiments.

## 4 Discussion

In Experiment 1, which explored the effects of the piezo pulses on the ooplasmic cell membrane, the survival rate of the oocytes decreased as the distance between the micropipette and the ooplasmic cell membrane decreased. Damage to the cell membrane greatly reduces subsequent cell viability; therefore, it is possible that the ooplasmic cell membrane was damaged when the piezo pulses were applied close to the cell membrane. In a preliminary experiment, PI staining was observed 24 h after drilling of the zona pellucida rather than immediately (data not shown); in Experiment 1, therefore, we evaluated damage to the ooplasmic cell membrane 24 h after drilling of the zona pellucida. The delayed observation of staining suggests that the effects of piezo pulses on the ooplasmic cell membrane are slight changes that become apparent only over time. We also found that cytoplasmic levels of Ca^2+^ in oocytes increased as the distance between the micropipette and the ooplasmic cell membrane decreased, further suggesting that the piezo pulses damaged the ooplasmic cell membrane, causing an influx of free Ca^2+^ions into the oocytes.

Piezo pulses are a type of ultrasound that causes shear forces in the fluid, as well as localized motions of the fluid called microstreaming [16]. It has been reported that microstreaming has a disruptive effect on the cell membranes of living organisms [17]. In the present study, therefore, the ooplasmic cell membrane may have been slightly damaged by microstreaming from the micropipette. The results of Experiment 1 indicate that the stronger the intensity of the piezo pulse, the greater the distance required for the oocyte to survive. Wang et al. [17] similarly reported that ultrasound intensity causes intensity‐dependent fluctuations in the plasma membrane potential of human leukemia cells, which they suggested was due to changes in the ion balance of the plasma membrane caused by loss of plasma membrane integrity. We also found that the levels of free Ca^2+^ ions in the oocyte fluctuated with the intensity of the piezo pulse, which may have led to an ionic imbalance in the ooplasmic cell membrane and reduced oocyte viability.

In Experiment 2, we examined the effect of sperm injection with the PB positioned at 2 or 4 o'clock (para‐PB piezo) on the integrity of the meiotic spindle. The spindle plays an important role in the normal arrangement and segregation of chromosomes during cell meiosis. Therefore, reducing the risk of the meiotic spindle damage during ICSI may improve the subsequent fertilization and developmental potential of the oocyte. A preliminary experiment found no significant differences in the meiotic spindle distribution area among bovine MII oocytes prepared by using different methods of cumulus cell removal (data not shown); therefore, we explored the effect of the injection site. To date, ICSI has been performed based on the assumption that the meiotic spindle is located near the PB, with sperm injection at 3 o'clock relative to the PB at the 12 or 6 o'clock [7]. However, Hewiston et al. reported that the PB and the meiotic spindle are not always located close together in mammalian oocytes [18]. Here, there was no difference in the integrity of the meiotic spindle between the conventional piezo group and the para‐PB piezo group, indicating that the PB and the meiotic spindle are not necessarily located together, consistent with Hewiston et al.'s report. In human MII oocytes, the position of the PB relative to the meiotic spindle is 90° is 7%–11% of cases [19, 20]. When the position of the PB relative to the meiotic spindle is more than 90°, fertilization rates are significantly lower due to spindle damage caused by the micropipette [8]. In Experiment 2, the integrity of the meiotic spindle was only 11.4% in the conventional piezo group, suggesting that the PB and meiotic spindle may not always be positioned adjacently in bovine MII oocytes, as in human MII oocytes. Observation of meiotic spindle movement during sham injection by the Oosight imaging system further revealed that, during micropipette injection, the meiotic spindle is pushed by the ooplasmic cell membrane and moves fluidly, escaping from the micropipette. Thus, we believe that even if the meiotic spindle is positioned at the micropipette injection site, it may escape from the micropipette tip because it is pushed aside by the ooplasmic cell membrane during injection.

Lastly, in Experiment 3, we determined the overall effectiveness of a piezo‐ICSI approach in which the PB is set at either the 2 or 4 o'clock position. We found that the oocyte survival rate 3 h after sperm injection was significantly higher in the para‐PB piezo group than in the conventional group. Improved oocyte survival rate is important because it increases the number of embryos available for implantation. The ooplasmic cell membrane is essential for the viability of the oocyte. It maintains a constant environment within the oocyte and selectively takes up substances for energy sources and pathways necessary for development. Chen et al. [21] reported that the low survival rate of oocytes in c‐ICSI is due to excessive damage to the ooplasmic cell membrane caused by aspiration. They also reported that piezo‐ICSI resulted in less damage to the ooplasmic cell membrane as compared with c‐ICSI [21]. Furthermore, disruption of the ooplasmic cell membrane by aspiration using laser or c‐ICSI methods damages not only the ooplasmic cell membrane but also the actin microtubules beneath the membrane [22]. In Experiment 3, we believe that damage to the ooplasmic cell membrane may have been reduced by perforating the widest position in the perivitelline space. In addition, there were no differences in blastocyst rate or chromosomal integrity between the para‐PB piezo group and the conventional piezo group. These results suggest that a new piezo‐ICSI method in which the PB is set at either the 2 or 4 o'clock position is useful and may be a way to increase the benefits of piezo‐ICSI.

In conclusion, our present study has demonstrated the effectiveness of a new piezo‐ICSI method in which zona drilling and sperm injection are performed at the para‐PB site (the widest position in the perivitelline space). Because piezo pulses may induce slight changes in the ooplasmic cell, our piezo‐ICSI method can improve oocyte survival by minimizing damage to the ooplasmic cell membrane during zona drilling and consequently increasing the number of embryos available for transfer.

## Ethics Statement

Institutional Review Board of the University of Utsunomiya (registration number: A15‐0012, A20‐0020).

## Conflicts of Interest

The authors declare no conflicts of interest.
